# Democratizing Neurorehabilitation: How Accessible are Low-Cost Mobile-Gaming Technologies for Self-Rehabilitation of Arm Disability in Stroke?

**DOI:** 10.1371/journal.pone.0163413

**Published:** 2016-10-05

**Authors:** Paul Rinne, Michael Mace, Tagore Nakornchai, Karl Zimmerman, Susannah Fayer, Pankaj Sharma, Jean-Luc Liardon, Etienne Burdet, Paul Bentley

**Affiliations:** 1 Division of Brain Sciences, Imperial College, London, United Kingdom; 2 Dept. of Bioengineering, Human Robotics Group, Imperial College, London, United Kingdom; 3 Institute of Cardiovascular Research, Royal Holloway University, London, United Kingdom; University of Florida, UNITED STATES

## Abstract

Motor-training software on tablets or smartphones (Apps) offer a low-cost, widely-available solution to supplement arm physiotherapy after stroke. We assessed the proportions of hemiplegic stroke patients who, with their plegic hand, could meaningfully engage with mobile-gaming devices using a range of standard control-methods, as well as by using a novel wireless grip-controller, adapted for neurodisability. We screened all newly-diagnosed hemiplegic stroke patients presenting to a stroke centre over 6 months. Subjects were compared on their ability to control a tablet or smartphone cursor using: finger-swipe, tap, joystick, screen-tilt, and an adapted handgrip. Cursor control was graded as: no movement (0); less than full-range movement (1); full-range movement (2); directed movement (3). In total, we screened 345 patients, of which 87 satisfied recruitment criteria and completed testing. The commonest reason for exclusion was cognitive impairment. Using conventional controls, the proportion of patients able to direct cursor movement was 38–48%; and to move it full-range was 55–67% (controller comparison: p>0.1). By comparison, handgrip enabled directed control in 75%, and full-range movement in 93% (controller comparison: p<0.001). This difference between controllers was most apparent amongst severely-disabled subjects, with 0% achieving directed or full-range control with conventional controls, compared to 58% and 83% achieving these two levels of movement, respectively, with handgrip. In conclusion, hand, or arm, training Apps played on conventional mobile devices are likely to be accessible only to mildly-disabled stroke patients. Technological adaptations such as grip-control can enable more severely affected subjects to engage with self-training software.

## Introduction

The most important intervention shown to improve physical function after stroke is repetitive, task-directed exercises, supervised by a physiotherapist, with higher intensity leading to faster and greater recovery[1]. In practice, access to physiotherapy is significantly limited by resource availability[2]. For example, 55% of UK stroke in-patients receive less than half the recommended physiotherapy time of 45 minutes per day[3].

One solution to inadequate physiotherapy is robotic technology, that enables patients to self-practice, with mechanical assistance, via interaction with adapted computer games. While a range of rehabilitation robotics have been marketed over the last decade, and shown to be efficacious[4], they are not widely used due to factors such as high-cost (typically, $10,000–100,000), cumbersome size, and restriction to patients with high baseline performance, and who have access to specialist rehabilitation centres[5].

An alternative approach to self-rehabilitation, are medical applications (Apps), or gaming software, run on mobile media devices e.g. tablets or smartphones[6, 7]. Because such devices are low-cost ($200–500), and ubiquitous, they have the potential to democratize computerized-physiotherapy, especially in under-resourced settings, e.g. chronically-disabled in the community. Furthermore, their portability enables home use, while their employment of motivational gaming strategies can potentiate high-intensity motor practice. Accordingly, increasing numbers of motor-training Apps for mobile devices have been commercialised in recent years, and clinical trials are under way[8, 9]. However, since these devices are designed for able-person use, it is questionable as to how well disabled people can access them, and engage meaningfully and repeatedly with rehabilitation software.

This study assesses the degree of motor interaction that can be achieved by hemiplegic stroke patients using four types of conventional hand-control methods (finger swipe, tap, joystick and tilt) for mobile devices. An adapted controller of the same mobile devices[10], whose materials cost ~$100, was evaluated alongside. Since the latter interface exploits the fact that handgrip is relatively spared in stroke hemiplegia[11], and is sensitive to subtle forces, we expected that this would increase the range of arm-disability severities able to achieve meaningful computer-game control. In order to assess motor control, with minimal cognitive confounding (given that many softwares also have cognitive demands), we used a simple 1-dimensional motor assessment for all controller types.

## Methods

### Participants

Consecutive stroke patients with arm weakness were screened over 6-months at Imperial College NHS Healthcare Trust Hyper-Acute Stroke Unit, within 2-weeks of presentation. We excluded patients with cognitive impairment (Mini-Mental State Examination <27), given their therapeutic gains from physiotherapy are generally poorer than those of cognitively-healthy individuals, and for ethical reasons. Other exclusion criteria were: 1) premorbid arm disability, or dependency (modified Rankin Score >2), 2) comprehension difficulty, 3) sensorimotor neglect (clinically, or >25% errors with star-cancellation test), 4) arm pain, 5) significant co-morbidities, 6) subsequent MRI failed to confirm stroke.

Patients’ arm disability was graded into one of three groups depending upon their score in the Upper Extremity section of the Short Fugl-Meyer Assessment (S-FM)^30^; FM): severe (0–4), moderate (5–8), and mild (9–12), where 12 is normal function. Arm power using MRC-grading, handgrip force using a manual dynamometer, handedness, mood and anxiety were also assessed. Recruited participants gave written and signed informed consent. Ethical approval was granted by the UK National Research Ethics Service, South East Coast Committee.

Subjects in the first three months were tested on their control of conventional game controllers; and in the second three months, on their control of the best-performing conventional game controller compared to a novel, adapted controller.

### Conventional Game-Control Assessment

**Fig 1:**
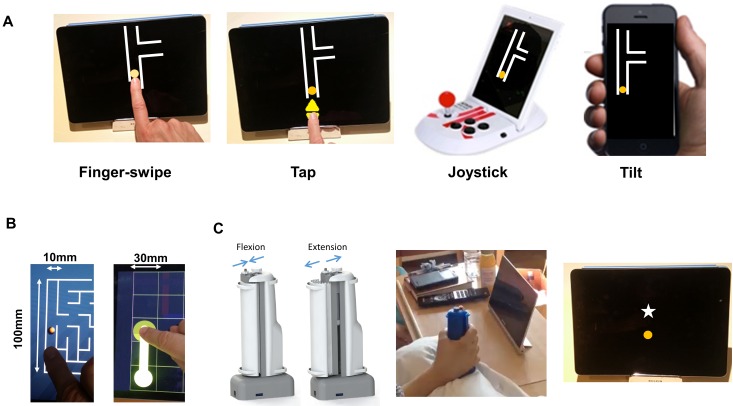
Control methods and devices trialled. Conventional control mechanisms were trialled using standard tablet and smartphone (**A, B**). Subjects were required only to move a cursor along a single vertical path, full-range, and then to an indicated vertical level (they were not tested on playing the underlying game). **B** shows software used for assessing swipe, with varying cursor size. There was no improvement in accessibility using a larger cursor. The novel control mechanism (**C**) is a wireless grip-force sensor that detects both finger-flexion and extension movements, the latter assisted by a fingerstrap holding the device within a partially-extended hand. Control software for **C** entailed moving a circle in a vertical plane towards a target star. Cursor and target stimuli dimensions and contrast are similar between all methods.

Subjects were asked to control a digital-screen cursor in the vertical plane using one of four hand-control methods employed by standard mobile or home-gaming devices: touch-screen swipe, tap, joystick and screen-tilt (Fig 1A). All subjects were tested on all four methods. For the first three methods, the cursor appeared on a 9.7-inch tablet; for tilt, a 3.5-inch smartphone. The joystick was integrated into a tablet-stand with which it interfaced (Atari Arcade Duo Powered). Devices were positioned to be most accessible and comfortable (e.g. in a stand or flat). Patients’ elbows could be supported by pillows.

Software used was a basic maze game that had similar graphics and functionality between all four types of control method (swipe: “4Kids Maze”, Gottaplay, 2014; tap, joystick: MazeCraze, Atari, 2012; tilt: “Tilt Mazes Lite”, Exact Magic Software, 2012). A maze was chosen in which one path ran approximately three-quarters the height of a landscape-orientated screen (10cm; or 7cm on a portrait-orientated smartphone). The cursor was positioned by the examiner at the bottom of the path so that the only movement possible was up or down (Fig 1B). The remainder of the screen could be occluded. Cursor size was 1 cm diameter for swipe, and 0.8 cm for other methods. For tapping, the cursor was controlled by 2 x 1.5cm up/down arrows.

**Fig 2:**
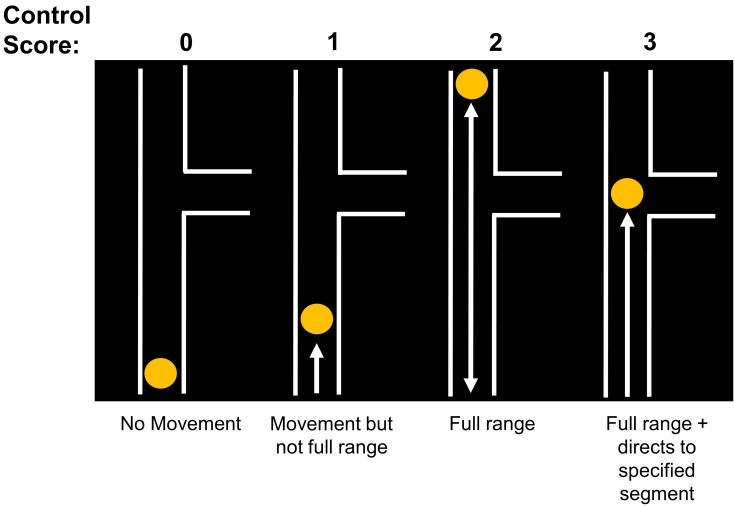
Cursor-control score. Subjects were asked to move the cursor three times up and down the longest vertical path, as well as to a position level with an indicated adjoining horizontal path.

Subjects were asked to move the cursor across the range of the vertical path in both directions. They then had to move the cursor towards an indicated section, level with where a horizontal path connected (without needing to move it sideways). Subjects were scored according to their ability to control the cursor as follows: 0: no movement of cursor; 1: moves cursor but not consistently across entire vertical range; 2: moves cursor consistently across entire vertical range in both directions, but cannot direct it to highlighted section; 3: moves cursor consistently across entire vertical range, and directs it to highlighted section (Fig 2). Three raters were used during the study, who achieved >95% inter-rater consistency in scoring by this method.

Subjects were allowed up to a minute per trial. Each trial was conducted three times, and the median control score recorded. Subjects were tested with their hemiplegic, and separately unaffected, arms. Control-method order was counterbalanced between subjects.

In order to assess the effect of cursor size and path direction, a substudy compared the swipe maze as described, with two alternative swipe software, that had larger cursors (2-3cm) and diagonal or horizontal path directions (Fig 1B; “FlowFree”, Big Duck Games, 2013; Traffic Controller 2, MindMender, 2013). Instructions, as above, were applied to these software.

### Adapted Handgrip Controller

Subjects were compared on their control of tablet swipe (as described above), versus an adapted, power-grip controller. This controller, designed for disability, utilises a patented force-sensing mechanism (flexible metal blade system) that allows functional, resistance-based training with high force sensitivity (0.1-50N) throughout the compliant range [10] (Fig 1C). The grip has adjustable compliance and girth, is portable and connects to a tablet wirelessly via Bluetooth. The handgrip also provides haptic (vibration) feedback, and senses inertial forces (accelerometer)–although these functions were not used in the current study.

Assessment of handgrip control used a software equivalent in cursor-movements and dimensions to that of the maze software. Handgrip force controlled a cursor that moved vertically; target positions were the upper and lower tablet-screen bounds, as well as a target star, the height of which was the same as the horizontal segment target in the conventional games. The star remained still or moved, the latter mode used for 2 –minute game play. Prior to assessment, the software is calibrated so that maximum cursor excursion is set to 70% of maximum voluntary contraction.

### Statistical Analysis

A generalised linear ordinal logistic regression model (Generalised Estimating Equation, SPSS V.22) estimated how movement control (0,1,2,3) was influenced by factors: control method (swipe, tap, joystick, tilt, grip), and arm type (hemiplegic, unaffected), with covariate of arm disability (severe, moderate, mild). An independent correlation matrix structure was selected.

### Handgrip-Control Sustained Performance

In a further cohort of 12 hemiplegic stroke patients, we assessed how performance accuracy using handgrip-control over 2 minutes of continuous game-play, related to arm disability. Accuracy was derived from root-mean square (RMS) distance-error between cursor and target (a moving star), calculated using a minimum moving error (MME) method, that reduces noise. At each time-point, RMS was calculated across a 15s window that it centred upon, and the lowest RMS error within this taken. The average across all such time-points was calculated, and regressed onto S-FM scores. These analyses were conducted in MATLAB (v2012).

## Results

**Fig 3:**
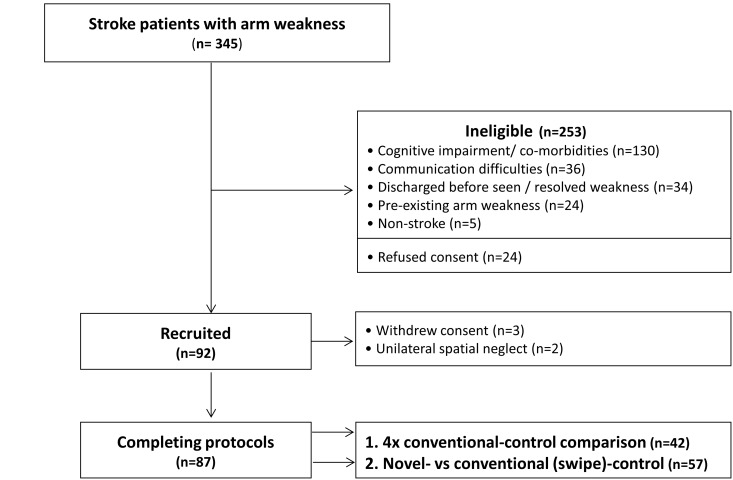
Recruitment flow diagram. This shows numbers of arm-paretic stroke patients screened, excluded and recruited, and reasons for exclusion.

345 patients with arm-weakness were screened, of which 92 were recruited and 87 completed the protocols (Fig 3). The principle reason for exclusion (51%) was cognitive impairment or physical comorbidities significant enough to make it impractical and unethical to test patients. Tested patients had less severe neurological deficits than those excluded (NIHSS 5 vs 9; p<0.05. Table 1). Of those recruited, most patients had mild, rather than moderate or severe, arm disability (60% vs 20% vs 20%).

**Table 1:** Patient demographics and baseline clinical characteristics.

	**Tested**	**Not Tested**
N	87	258
Age / yrs	65 (55–75)	72 (64–85)
Males / %	57	56
NIHSS–overall/42	5 (2–6)	9 (4–14)*
Hospital Anxiety and Depression Scale—/42	3 (1–3)	4 (1–10)
Edinburgh Handedness Inventory	100 (100–100)	-
**Arm Specific Tests**	**Weak Hand**	
Plegic hand-side	Right-hand: 42%	
Short Fugl Meyer arm function /12	8 (6–11)	
Hand Section Fugl Meyer /14	8 (2–13)	
Grip Force /Kg	13 (2–22)	

Median (interquartile range).

* p<0.05.

### Conventional-Control Comparison

**Fig 4:**
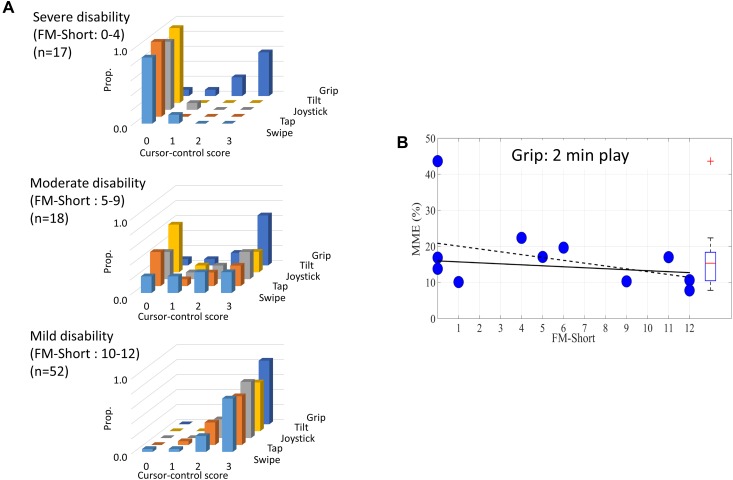
Control ability using conventional versus novel controllers. **A**: Proportions of patients achieving each level of cursor control (0–3) for each of the four conventional, and one novel (grip), control mechanism. Results are stratified according to severity of arm weakness (using Short-Fugl-Meyer score of the arm). **B**: Performance error on 2-minute tracking task controlled by grip-control, plotted against arm disability. A small trend towards less error with greater ability is non-significant whether or not the one outlier is included (dashed-line) or not (continuous-line) (p>0.1 for both)–indicating that tracking accuracy is largely independent of standard arm-function scores.

Control scores in the hemiplegic arm were strongly affected by arm disability level (Wald chi(1) = 44.5, p<0.001), with the proportion being able to use at least one conventional control to direct a cursor to target (score = 3) being 90% for mildly disabled, 36% for moderately disabled, and 0% for severely disabled (Fig 4A). However, control scores did not differ significantly between the four conventional control types (chi2(3) = 2.7; p>0.1), with the proportion of patients achieving a score of 3 being 48%, 45%, 38% and 38%, for swipe, joystick, tap and tilt, respectively. There was no control type x disability interaction (chi2(3) = 1.5; p>0.1). There was also no difference in control scores comparing the three swipe software that varied in cursor-size (1–3 cm) and path-direction (n = 27; chi2(2) = 0.5; p>0.1).

Influence of arm (hemiplegic vs unaffected) on device control was seen as an interaction with disability (chi2(1) = 15.4; p<0.001), reflecting significantly poorer control using hemiplegic than unaffected arm in severely (p<0.001) and moderately disabled (p<0.05) subjects, but not mildly disabled. However, control was also poorer in the *unaffected* arm in severely disabled patients (score: 2.45), compared to unaffected arm of moderate and mild patients (score: 2.95–3; chi2(1) = 7.5; p<0.01).

### Novel-Handgrip vs. Conventional-Control Comparison

Compared to finger-swipe–the best conventional control–the novel handgrip controller resulted in superior software control (chi2(1) = 20.2; p<0.001). The proportion achieving control-score of 3 was 48% for swipe vs 75% for grip; whilst the proportion achieving control-score of 2 or 3 was 67% for swipe and 93% for grip (all values quoted are using the hemiplegic arm). The superiority of grip over swipe was greater at higher levels of disability (device x disability interaction: chi2(1) = 10.2, p<0.01), e.g. in severely disabled subjects, control-score 3 was achieved in 58% with grip, versus 0% with swipe (Fig 4A).

Performance accuracy for 2-minute game play (measured as minimum moving error, MME) using grip control was minimally affected by disability severity (correlation with S-FM being non-significant: r = -0.27; p = 0.21). For example, performance in 3/5 patients with severe disability was within the range of mildly disabled patients (Fig 4B). An example of a patient, who scores 0 on tablet swipe, and then successfully controls a visuomotor tracking software with their severely disabled arm, using handgrip control, is shown in S1 Video.

## Discussion

The first part of our study indicates that standard use of everyday mobile devices for arm physical therapy in stroke, is likely to be limited. Less than half of recruited subjects could direct a cursor using conventional tablet or smartphone mechanisms, with their paretic arm. Furthermore, patients in severe- or moderate-disability bands–for whom physiotherapy requirements and potential gains are higher—directed control in 0% or ~30%, respectively. Clinical trials looking at the potential benefits of tablet-based arm-training software, using standard controls[6, 9], will therefore be restricted to mildly-disabled patients.

The accessibility of mobile devices for arm training is likely to be even lower than that estimated here for several reasons. Firstly, we excluded 75% of hemiplegic patients, for reasons such as dementia, yet such subjects had higher disability than those tested. Given a steep fall-off in control as disability increased, and given this group’s poorer cognition and co-morbidities, the excluded majority would probably be far less capable than we found. Furthermore, our motor test was limited to a single, one-dimensional movement, whereas training software typically entails practice for many minutes, more demanding tasks, in two dimensions etc.–all of which are likely to reduce successful performance.

It is likely that software factors, e.g. task simplicity, cursor size, in addition to interface mechanism, influence control[12]. However, we deliberately chose a task that had minimal cognitive demands, high-contrast graphics, and did not time-pressure patients. The fact that there were no performance differences between three types of swipe software (one of which is designed for arm rehabilitation, the other using a larger 3cm cursor) suggests that task-type or graphics are not major determinants for software inaccessibility. While even larger screen targets than those tested here[9], could enable more patients to engage, the range and utility of potential exercises is likely to decrease as the target size increases. Moreover, gross tapping is a relatively uncontrolled movement that could be achieved by truncal or flailing movements that are not the games’ intended purpose.

The possibility that cognitive or visuoperceptual impairments, commonly found in stroke[13], may have reduced performance ability is discounted by the finding that patients achieved good control, with all methods, using their non-plegic hand. Whilst severely disabled patients did show mild impairment using their non-plegic hand, this is likely to reflect an ipsilesional motor deficit[14], rather than because of cognitive factors, given that cognitive impairment was an exclusion criterion.

In the only other study of its kind, 20 stroke patients were tested with a tablet using swipe and tap control[12]. Of these, 7/20 were able to swipe consistently, while 15/20 were able to complete a tapping game performed twice. While the latter figure suggests a greater potential for motor-training on tablets than found here, tapping accuracy in that study was only 50%. Furthermore, the test population was disproportionately mild, being a convenience sample, and excluded patients with severe hand weakness. Consequently, arm ability in that study relative to healthy controls was 92% (using the Fugl-Meyer scale), as opposed to 57% in our consecutively-sampled series, that is likely to be more representative.

In comparison to the best-performing conventional control method (i.e. swipe), we found that a simple, economical adaptation to mobile devices can significantly increase accessibility, particularly in more severely affected patients. A handgrip controller increased the proportion of *all* patients able to achieve cursor control by ~50% (relative to swipe); and enabled more than half of severely disabled patients to engage with tablet software, as compared to 0% using any other method.

The reason why handgrip enables superior control compared to other methods most likely arises from the differential pattern of arm weakness found after stroke. Hence gross-grasp is one of the least affected movements, whereas individuated finger movements, wrist extension and supination–required for swipe, joystick or tilt—are more impaired[11]. Furthermore, the fact that the majority of severely hemiplegic patients were not just able to move the cursor across the entire range, but were able to *direct* cursor control, underlies a previous finding, that fine-grip control may be independent of grip strength[15]. This is also apparent during the demanding 2-minute tracking task, in which handgrip accuracy of severe hemiplegics was similar to that of more able patients. The grip controller enables this fine control by calibrating software to patients’ maximum strength, and sensing forces across a wide range.

While we have shown that grip-control, relative to other control methods, increases the proportion of patients able to engage with rehabilitation software, our study does not address the question of whether such repetitive practice would lead to functional benefits. Although power-grip is one of the least affected arm functions following stroke [11], there are multiple aspects of grip control that are deranged after stroke, e.g. smoothness, force distribution and grip-release[15–18], even when other aspects e.g. tracking accuracy, are performed well. Software could therefore be designed to train these more affected aspects of grip control, as well as to encourage finger-extension over flexion. For example software can be calibrated so that a patient’s grip-neutral position is matched to a cursor location at screen bottom, and the only hand movements able to move the cursor upwards are finger extension (assisted by a strap holding the controller in the hand). A related question is whether repetitive exercise of a single action e.g. graduated grip flexion-extension, could confer functional benefits beyond those of the action practiced. At least five trials of robotic hand-trainers in stroke have shown that frequent hand-training e.g. grasping or finger exercises, result in functional gains not only in the hand, but also in more proximal arm movements[19–22], that may reflect automatic upper-arm posturing during distal actions such as gripping, and generalisation of motor learning[23]. Whether this result could be repeated on a larger scale, in patients’ homes, using portable electronic aids such as that tested here, are relevant future research directions.

The hand-grip interface described here is one example of several portable arm-rehabilitation innovations developed in recent years, commercially available at relatively low-cost ($500–3000). The MusicGlove^®^ for instance is a wearable sensor that interfaces with PC-based software, designed for home-training of grip and individuated finger movements[24]. The Tyromotion Pablo^®^ is an isometric powergrip sensor that interfaces via a wire with desktop-PC software. Other devices that interact with computer software are designed for wrist or upper-arm training, e.g. the Kinestica Bimeo^®^, as well as the digital handgrip tested in the current study, that has a separate accelerometer capability (not assessed here). Future studies will be required to determine the range of patient abilities for whom each device may be a useful training aid. We would hypothesise from the profile of arm disability after stroke[11], that the power-grip tested here will be more suited to patients with severe disability, whereas aids training individual finger movements, or anti-gravity proximal arm movements, would be more relevant to patients with milder disability.

In summary, our study highlights a major limitation of everyday mobile technologies for arm training after stroke, and suggests one low-cost method by which restricted interaction can be overcome. Whether or not improving access to physiotherapy-based computer games translates into increased self-training by patients, and ultimately functional benefits, are questions for future research.

## Supporting Information

S1 File Raw-data of conventional versus novel control experiment (relates to Fig 4). (XLSX)

S1 Video Demonstration of a severely hemiplegic patient attempting tablet control using swipe, and novel hand-grip controller. The patient’s only recorded arm movements are flickers of finger flexors (FM-S 1/12). The patient was able to successfully engage with a visuo–motor tracking software using the grip controller tested in this study.
(MP4)

## References

[1] Lohse KR, Lang CE, Boyd LA. Is more better? Using metadata to explore dose-response relationships in stroke rehabilitation. Stroke. 2014;45(7):2053–8. 10.1161/STROKEAHA.114.004695 24867924 PMC4071164

[2] Bernhardt J, Chan J, Nicola I, Collier JM. Little therapy, little physical activity: rehabilitation within the first 14 days of organized stroke unit care. J Rehabil Med. 2007;39(1):43–8. 10.2340/16501977-0013 . 17225037

[3] NICE. National Institute of Clinical Excellence: CG 162: Stroke rehabilitation: costing report. 2013. Available: https://www.nice.org.uk/guidance/cg162/resources/cg162-stroke-rehabilitation-costing-report2.

[4] Norouzi-Gheidari N, Archambault PS, Fung J. Effects of robot-assisted therapy on stroke rehabilitation in upper limbs: systematic review and meta-analysis of the literature. J Rehabil Res Dev. 2012;49(4):479–96. 10.1682/JRRD.2010.10.0210 . 22773253

[5] Wagner TH, Lo AC, Peduzzi P, Bravata DM, Huang GD, Krebs HI, et al An economic analysis of robot-assisted therapy for long-term upper-limb impairment after stroke. Stroke. 2011;42(9):2630–2. 10.1161/STROKEAHA.110.606442 21757677 PMC4445835

[6] Thomson K, Pollock A, Bugge C, Brady MC. Commercial gaming devices for stroke upper limb rehabilitation: a survey of current practice. Disabil Rehabil Assist Technol. 2015:1–8. 10.3109/17483107.2015.1005031 . 25634339

[7] Thomson K, Pollock A, Bugge C, Brady M. Commercial gaming devices for stroke upper limb rehabilitation: a systematic review. Int J Stroke. 2014;9(4):479–88. 10.1111/ijs.12263 . 24661797

[8] Rand D, Zeilig G, Kizony R. Rehab-let: touchscreen tablet for self-training impaired dexterity post stroke: study protocol for a pilot randomized controlled trial. Trials. 2015;16:277 10.1186/s13063-015-0796-9 26081864 PMC4476080

[9] Saposnik G, Chow CM, Gladstone D, Cheung D, Brawer E, Thorpe KE, et al. iPad technology for home rehabilitation after stroke (iHOME): a proof-of-concept randomized trial. Int J Stroke. 2014;9(7):956–62. 10.1111/ijs.12328 . 25042159

[10] Mace M, Rinne P, Liardon J, Bentley P, Burdet E, editors. Comparison of flexible and rigid hand-grip control during a feed-forward visual tracking task. Rehabilitation Robotics (ICORR), 2015 IEEE International Conference; 2015.

[11] Raghavan P. The nature of hand motor impairment after stroke and its treatment. Curr Treat Options Cardiovasc Med. 2007;9(3):221–8. 10.1007/s11936-007-0016-3 . 17601386

[12] Kizony R, Zeilig G, Dudkiewicz I, Schejter-Margalit T, Rand D. Tablet Apps and Dexterity: Comparison Between 3 Age Groups and Proof of Concept for Stroke Rehabilitation. J Neurol Phys Ther. 2016;40(1):31–9. 10.1097/NPT.0000000000000110 . 26630324

[13] Rinne P, Hassan M, Goniotakis D, Chohan K, Sharma P, Langdon D, et al Triple dissociation of attention networks in stroke according to lesion location. Neurology. 2013;81(9):812–20. 10.1212/WNL.0b013e3182a2ca34 . 23902704 PMC3908461

[14] Noskin O, Krakauer JW, Lazar RM, Festa JR, Handy C, O'Brien KA, et al Ipsilateral motor dysfunction from unilateral stroke: implications for the functional neuroanatomy of hemiparesis. J Neurol Neurosurg Psychiatry. 2008;79(4):401–6. 10.1136/jnnp.2007.118463 . 17635970

[15] Lindberg PG, Roche N, Robertson J, Roby-Brami A, Bussel B, Maier MA. Affected and unaffected quantitative aspects of grip force control in hemiparetic patients after stroke. Brain Res. 2012;1452:96–107. 10.1016/j.brainres.2012.03.007 . 22464180

[16] Naik SK, Patten C, Lodha N, Coombes SA, Cauraugh JH. Force control deficits in chronic stroke: grip formation and release phases. Exp Brain Res. 2011;211(1):1–15. 10.1007/s00221-011-2637-8 . 21448576

[17] Beebe JA, Lang CE. Active range of motion predicts upper extremity function 3 months after stroke. Stroke. 2009;40(5):1772–9. 10.1161/STROKEAHA.108.536763 19265051 PMC2718540

[18] Jarrassé N, Kühne M, Roach N, Hussain A, Balasubramanian S, Burdet E, et al Analysis of grasping strategies and function in hemiparetic patients using an instrumented object. IEEE Int Conf Rehabil Robot. 2013;2013:6650379 10.1109/ICORR.2013.6650379 . 24187198

[19] Balasubramanian S, Klein J, Burdet E. Robot-assisted rehabilitation of hand function. Curr Opin Neurol. 2010;23(6):661–70. 10.1097/WCO.0b013e32833e99a4 . 20852421

[20] Susanto EA, Tong RK, Ockenfeld C, Ho NS. Efficacy of robot-assisted fingers training in chronic stroke survivors: a pilot randomized-controlled trial. J Neuroeng Rehabil. 2015;12:42 10.1186/s12984-015-0033-5 25906983 PMC4422529

[21] Krebs HI, Volpe BT, Williams D, Celestino J, Charles SK, Lynch D, et al Robot-aided neurorehabilitation: a robot for wrist rehabilitation. IEEE Trans Neural Syst Rehabil Eng. 2007;15(3):327–35. 10.1109/TNSRE.2007.903899 17894265 PMC2733849

[22] Lambercy O, Dovat L, Yun H, Wee SK, Kuah CW, Chua KS, et al Effects of a robot-assisted training of grasp and pronation/supination in chronic stroke: a pilot study. J Neuroeng Rehabil. 2011;8:63 10.1186/1743-0003-8-63 22087842 PMC3280186

[23] Dipietro L, Krebs HI, Volpe BT, Stein J, Bever C, Mernoff ST, et al Learning, not adaptation, characterizes stroke motor recovery: evidence from kinematic changes induced by robot-assisted therapy in trained and untrained task in the same workspace. IEEE Trans Neural Syst Rehabil Eng. 2012;20(1):48–57. 10.1109/TNSRE.2011.2175008 22186963 PMC4687974

[24] Friedman N, Chan V, Reinkensmeyer AN, Beroukhim A, Zambrano GJ, Bachman M, et al Retraining and assessing hand movement after stroke using the MusicGlove: comparison with conventional hand therapy and isometric grip training. J Neuroeng Rehabil. 2014;11:76 10.1186/1743-0003-11-76 24885076 PMC4022276

